# A Novel Germanium-Around-Source Gate-All-Around Tunnelling Field-Effect Transistor for Low-Power Applications

**DOI:** 10.3390/mi11020164

**Published:** 2020-02-03

**Authors:** Ke Han, Shanglin Long, Zhongliang Deng, Yannan Zhang, Jiawei Li

**Affiliations:** School of Electronic Engineering, Beijing University of Posts and Telecommunications, Haidian District, Beijing 100876, China; longshanglin@bupt.edu.cn (S.L.); dengzhl@bupt.edu.cn (Z.D.); murmures@bupt.edu.cn (Y.Z.); jiawei@bupt.edu.cn (J.L.)

**Keywords:** band-to-band tunnelling (BTBT), tunnelling field-effect transistor (TFET), germanium-around-source gate-all-around TFET (GAS GAA TFET), average subthreshold swing

## Abstract

This paper presents a germanium-around-source gate-all-around tunnelling field-effect transistor (GAS GAA TFET). The electrical characteristics of the device were studied and compared with those of silicon gate-all-around and germanium-based-source gate-all-around tunnel field-effect transistors. Furthermore, the electrical characteristics were optimised using Synopsys Sentaurus technology computer-aided design (TCAD). The GAS GAA TFET contains a combination of around-source germanium and silicon, which have different bandgaps. With an increase in the gate-source voltage, band-to-band tunnelling (BTBT) in silicon rapidly approached saturation since germanium has a higher BTBT probability than silicon. At this moment, germanium could still supply current increment, resulting in a steady and steep average subthreshold swing $\left(SS_{\mathrm{AVG}}\right)$ and a higher ON-state current. The GAS GAA TFET was optimised through work function and drain overlapping engineering. The optimised GAS GAA TFET exhibited a high ON-state current ($I_{\mathrm{ON}}$) (11.9 μA), a low OFF-state current ($I_{\mathrm{OFF}}$) ($2.85\times 10^{-9}$μA), and a low and steady $SS_{\mathrm{AVG}}$ (57.29 mV/decade), with the OFF-state current increasing by $10^{7}$ times. The GAS GAA TFET has high potential for use in low-power applications.

## 1. Introduction

Owing to rapid advances in semiconductor device technology, fifth-generation communication devices, wearable devices, Internet of Things, and numerous information technology devices have been developed. In the scaling of semiconductor devices to the nanoscale regime in accordance with Moore’s law, power consumption is one of the major impediments. Decreasing the supply voltage is an effective way to reduce power consumption. However, in conventional metal-oxide-semiconductor field-effect transistors (MOSFETs), subthreshold swing (SS) is limited to 60 mV/decade (SS=(kT/q)×ln10) at room temperature. This limitation prevents the supply voltage from being reduced at the same pace as the scaling of the physical dimensions of semiconductor devices [1,2,3,4,5]. To overcome this problem, researchers have been studying devices with a steep SS. Owing to their conduction mechanisms, such as impact ionization and band-to-band tunnelling (BTBT), differing from that of conventional MOSFETs, the ionization MOS (I-MOS), which is based on impact ionization, and tunnelling field-effect transistor (TFET), which is based on BTBT, can achieve the SS, lower than 60 mV/decade. Therefore, both these transistors have attracted considerable research interest. However, I-MOS is not suitable for low-power applications owing to its high breakdown voltage [6,7,8,9]. By contrast, TFETs provide a steeper SS, a lower OFF-state current ($I_{\mathrm{OFF}}$) and a lower supply voltage compared to conventional MOSFETs [10,11,12,13,14,15] and are suitable for low-power applications.

As mentioned, TFETs are based on BTBT conduction mechanism. This implies that the flow of drain current in n-channel-TFET occurs through tunnelling of charge carriers from the valence band of the source to the conduction band of the channel region [16]. Consequently, TFETs have a low $I_{\mathrm{OFF}}$ and can achieve a sub-60 mV/decade SS. The task of reducing the SS has drawn considerable attention and many studies have been conducted in this regard. The minimum point subthreshold swing ($SS_{\mathrm{MIN}}$) was 5 mV/decade in [17] and 11 mV/decade in [18]. However, focusing only on reducing $SS_{\mathrm{MIN}}$ is insufficient. In low-power applications, the average subthreshold swing ($SS_{\mathrm{AVG}}$) is far more significant than $SS_{\mathrm{MIN}}$ [3]. $SS_{\mathrm{AVG}}$ is generally calculated as

$$SS_{\mathrm{AVG}}=\frac{V_{T}-V_{\mathrm{OFF}}}{log\left(I_{T}\right)-log\left(I_{\mathrm{OFF}}\right)}\;$$

where $V_{T}$ is the threshold voltage, $I_{T}$ is the current at $V_{T}$, and $V_{\mathrm{OFF}}$ is the gate voltage in the OFF-state. Unfortunately, in conventional Si TFETs, as the gate-source voltage increases, BTBT rapidly approaches saturation, which causes $SS_{\mathrm{AVG}}$ to increase dramatically. Hence, unlike conventional MOSFETs where $SS_{\mathrm{AVG}}$ is approximately equal to $SS_{\mathrm{MIN}}$, the value of $SS_{\mathrm{AVG}}$ in conventional Si TFETs is always considerably larger than $SS_{\mathrm{MIN}}$ [19,20]. However, $SS_{\mathrm{AVG}}$ dramatically increases as $V_{\mathrm{gs}}$ increases, resulting in $SS_{\mathrm{AVG}}$ and $SS_{\mathrm{MIN}}$ differing considerably and $SS_{\mathrm{AVG}}$ becoming unsteady. This is a disadvantage of conventional Si TFETs. Owing to the large bandgap and carrier mass of the silicon material, conventional Si TFETs have another disadvantage: A low ON-state current ($I_{\mathrm{ON}}$) [21,22,23]. To overcome these disadvantages, significant research has been conducted and several device structures have been proposed. It has been shown that the use of a narrow-bandgap material such as germanium(Ge) as the source base to implement a heterojunction structure could lead to a considerably higher $I_{\mathrm{ON}}$ [24,25,26,27,28,29]. In particular, owing to its small screening length and high gate controllability, TFETs with a gate-all-around (GAA) structure have been extensively studied for achieving a high $I_{\mathrm{ON}}$ [2,18,19]. Conventional Si TFETs exhibit a steep $SS_{\mathrm{MIN}}$ and a low $I_{\mathrm{ON}}$ [30]. While heterojunction TFETs exhibit a high $I_{\mathrm{ON}}$, their $SS_{\mathrm{MIN}}$ and $SS_{\mathrm{AVG}}$ need to be improved [28]. Thus, further research on TFET devices is required to achieve a steady $SS_{\mathrm{AVG}}$ and a higher $I_{\mathrm{ON}}$.

In this paper, we propose a novel germanium-around-source gate-all-around TFET (GAS GAA TFET). In this device, the source is surrounded by germanium and a germanium-silicon heterojunction is formed at source. The GAS GAA TFET is expected to have a high $I_{\mathrm{ON}}$ and a steady $SS_{\mathrm{AVG}}$ and suppress the SS degradation behaviour. The characteristics of the device were investigated in detail to evaluate its capability for low-power applications.

## 2. Device Structures and Simulation Methods

**Figure 1:**
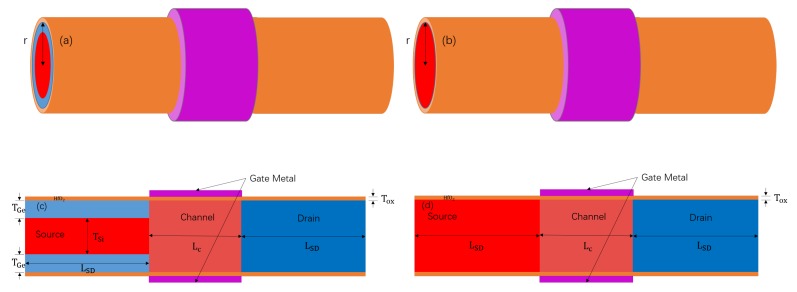
Schematics of the (**a**) germanium-around-source (GAS) gate-all-around (GAA) tunnelling field-effect transistor (TFET) and (**b**) Si GAA TFET and Ge-source GAA TFET. Cross-sectional views of the (**c**) GAS GAA TFET and (**d**) Si GAA TFET and Ge-source GAA TFET.

Figure 1a shows a schematic of the proposed GAS GAA TFET with a channel radius (*r*) of 12 nm, and Figure 1c depicts a cross-sectional view of the device. $T_{\mathrm{Ge}}$ is the thickness of the germanium layer, which surrounds silicon in the source. The channel and drain of the device were made of silicon. The gate dielectric material was hafnium oxide (${\mathrm{HfO}}_{2}$) and the thickness ($T_{\mathrm{ox}}$) of the oxide layer was 2 nm. The doping concentrations of the source, channel, drain, and around-source germanium were $5\times 10^{19}\;$cm${}^{-3}$ (p-type), $1\times 10^{15}\;$cm${}^{-3}$ (p-type), $1\times 10^{17}\;$cm${}^{-3}$ (n-type), and $5\times 10^{19}\;$cm${}^{-3}$ (p-type), respectively. The channel length, source length, and drain length were 30, 40, and 40 nm, respectively. All design parameters are presented in Table 1. Figure 1b shows a schematic of the control groups silicon gate-all-around TFET (Si GAA TFET) and germanium-based-source gate-all-around TFET (Ge-source GAA TFET), and Figure 1d presents a cross-sectional view of the control groups. The distinction between the Si GAA TFET and the Ge-source GAA TFET lies in the material of the source. The source material of the former is silicon, while that of the latter is germanium. The Si GAA TFET and Ge-source GAA TFET are identical to the GAS GAA TFET, except for the source.

All simulation results in this study were obtained using the nonlocal BTBT model, Shockley–Read–Hall recombination model, bandgap narrowing model, and doping dependence mobility model in Synopsys Sentaurus TCAD. The parameters used to calibrate the nonlocal BTBT model were $A=4\times 10^{14}\;$cm${}^{-3}$s${}^{-1}$, and $B=19\times 10^{6}\;$V/cm for silicon and $A=1.46\times 10^{17}\;$cm${}^{-3}$s${}^{-1}$, and $B=3.59\times 10^{6}\;$V/cm for germanium [1].

## 3. Simulation Results and Discussion

### 3.1. Thickness of Germanium ($T_{\mathrm{Ge}}$)

**Figure 2:**
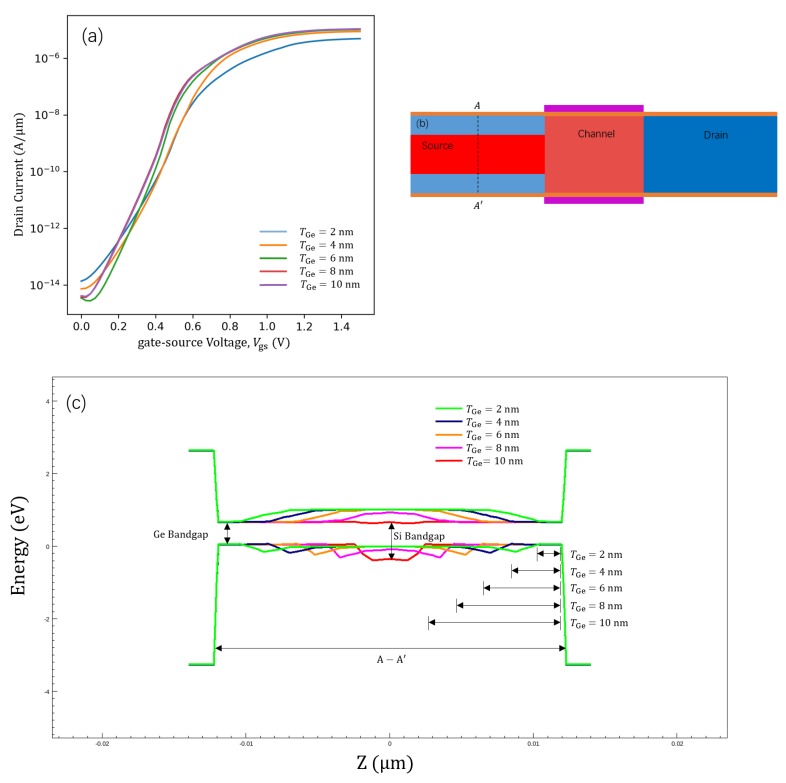
(**a**) $I_{D}-V_{\mathrm{gs}}$ transfer characteristics for different $T_{\mathrm{Ge}}$ values from 2 to 10 nm. (**b**) Cross-sectional view of the GAS GAA TFET; AA’ represents a cut line. (**c**) Energy band diagram for the GAS GAA TFET along the cut-line AA’ shown in (**b**).

Figure 2a shows the $I_{D}-V_{\mathrm{gs}}$ transfer characteristics of the proposed GAS GAA TFET for different $T_{\mathrm{Ge}}$ values. The $T_{\mathrm{Ge}}$ values considered were 2, 4, 6, 8, and 10 nm, and the gate material’s work function was 4.53 eV. The transfer characteristics show that $I_{\mathrm{ON}}$ increases with $T_{\mathrm{Ge}}$ since the effective tunnelling barrier width decreases with an increase in $T_{\mathrm{Ge}}$ [31]. For low-voltage operation, germanium (bandgap = 0.66 eV) showed a higher BTBT rate than silicon (bandgap = 1.12 eV). The internal mechanism responsible for the GAS GAA TFET performance improving with an increase in $T_{\mathrm{Ge}}$ from 2 to 10 nm can be inferred from the energy band diagrams shown in Figure 2c. The energy band diagrams are for a lateral-section of the source corresponding to the cut line A-A’ in Figure 2b. In Figure 2c, the bandgaps of germanium and silicon are shown; germanium has a narrower bandgap than silicon. As $T_{\mathrm{Ge}}$ changed from 2 to 10 nm, the area of the narrow bandgap material increased in the source. The BTBT probability ($T_{\mathrm{WKB}}$) is given by the Wentzel–Kramers–Brillouin (WKB) approximation ($T_{\mathrm{WKB}}≃exp\left(\frac{-4\lambda \sqrt{2m^{*}}\sqrt[{3}]{E_{g}}}{3q\hbar +\Delta \phi }\right)$, and $I_{\mathrm{ON}}$ is correlated with $T_{\mathrm{WKB}}$ [2]. Hence, an increase in $T_{\mathrm{Ge}}$ from 2 to 10 nm leads to germanium becoming the main semiconductor material. In the formula for $T_{\mathrm{WKB}}$, $E_{g}$ is the bandgap of the main semiconductor material in the device, and germanium becoming the main semiconductor material reduces $E_{g}$, which improves $I_{\mathrm{ON}}$.

### 3.2. Effect of Germanium-Around-Source (GAS)

**Figure 3:**
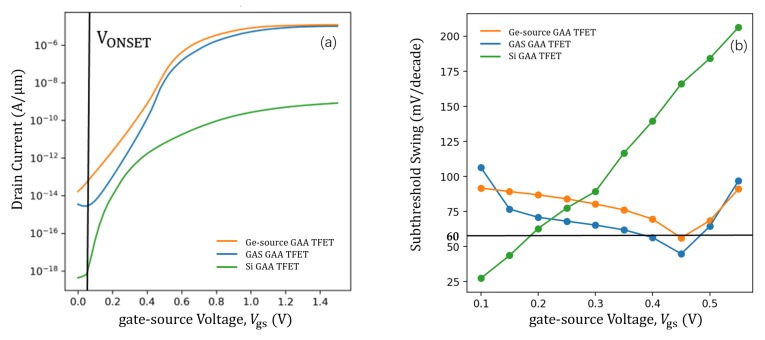
(**a**) $I_{D}-V_{\mathrm{gs}}$ transfer characteristics and (**b**) $\mathrm{SS}-V_{\mathrm{gs}}$ curves for GAS GAA TFET, Ge-source GAA TFET, and Si GAA TFET.

**Figure 4:**
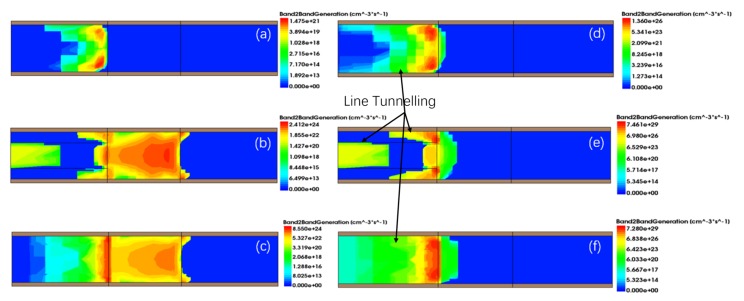
Two-dimensional band-to-band tunnelling (BTBT) generation contour plots for the (**a**,**d**) Si GAA TFET, (**b**,**e**) GAS GAA TFET, and (**c**,**f**) Ge-source GAA TFET. Panels (**a**–**c**) are for $V_{\mathrm{DS}}$= 1 V and $V_{\mathrm{gs}}$ = 0.1 V and panels (**d**–**f**) are for $V_{\mathrm{DS}}$ = 1 V and $V_{\mathrm{gs}}$ = 0.5 V.

**Figure 5:**
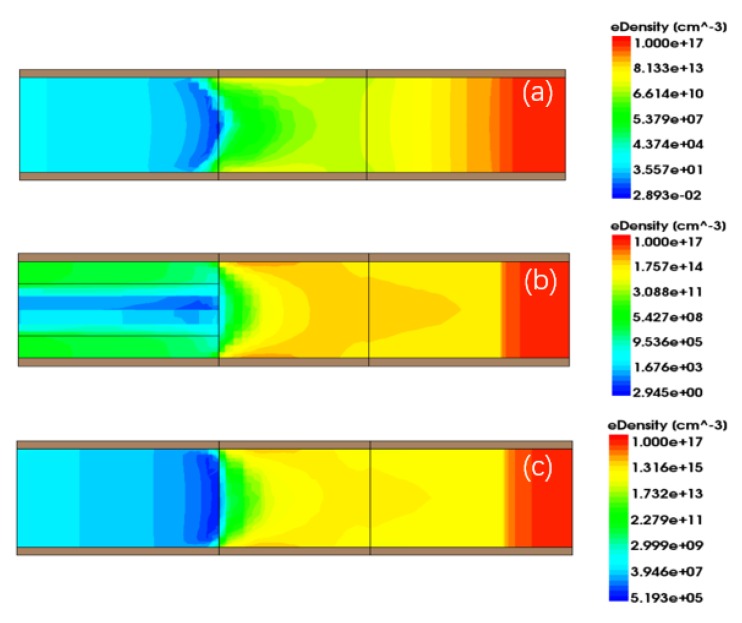
Two-dimensional electron density contours for the (**a**) Si GAA TFET, (**b**) GAS GAA TFET, and (**c**) Ge-source GAA TFET at $V_{\mathrm{DS}}$ = 1 V and $V_{\mathrm{gs}}$ = 0.5 V in thermal equilibrium.

Figure 3a shows a comparison of the transfer characteristics of the proposed GAS GAA TFET for $T_{\mathrm{Ge}}$ = 6 nm with the Si GAA TFET and Ge-source GAA TFET. For a fair comparison, the gate material work function for the Si GAA TFET was tuned to 4.1 eV to obtain approximately the same onset voltage ($V_{\mathrm{ONSET}}$) as the GAS GAA TFET and Ge-source GAA TFET; the onset voltage is the voltage after which the drain current increases exponentially with the gate voltage as shown in Figure 3 [1]. The gate material work function for the GAS GAA TFET and Ge-source GAA TFET was 4.53 eV. The GAS GAA TFET exhibited a steady and steeper $SS_{\mathrm{AVG}}$ than the Si GAA TFET and Ge-source GAA TFET, and a higher ON-state current than the Si GAA TFET. Here, the threshold voltage ($V_{T}$) was defined as the voltage where the current increased by a factor of $10^{7}$. $I_{T}$ and $V_{T}$ of the GAS GAA TFET, Si GAA TFET, and Ge-source GAA TFET were $10^{-7}$ A and 0.579 V, $10^{-11}$ A and 0.537 V, and $10^{-6}$ A and 0.663 V, accordingly. Moreover, $SS_{AVG}$ for these devices was 65, 68.71, and 83.71 mV/decade, separately. Figure 3b shows a comparison of the SS as a function of the gate-source voltage ($V_{\mathrm{gs}}$) among the GAS GAA TFET, Si GAA TFET, and Ge-source GAA TFET. Evidently, the SS of the GAS GAA TFET is steadier than that of the Si GAA TFET in a wide voltage range, and it is lower than those of the Si GAA TFET and Ge-source GAA TFET for most of the $V_{\mathrm{gs}}$ range considered. Since the trends of the GAS GAA TFET and the Ge-source GAA TFET curves are similar, we calculated their variances. In the range of $V_{\mathrm{gs}}$ = 0.15 V to 0.5 V, the variance of the GAS GAA TFET is 81.92 (mV/decade)${}^{2}$ and the variance of the Ge-source GAA TFET is 108.01 (mV/decade)${}^{2}$. Moreover, it proves that GAS GAA TFET is steadier than Ge-source GAA TFET in a wide voltage. As shown in the BTBT generation contour plot in Figure 4, the BTBT electron generation rate varies with $V_{\mathrm{gs}}$. Since the germanium-around-source structure involves a combination of silicon and germanium, at $V_{\mathrm{gs}}$ = 0.1 V and $V_{\mathrm{DS}}$ = 1 V, BTBT generation for the GAS GAA TFET in Figure 4b is greater than that for the Si GAA TFET in Figure 4a and less than that for the Ge-source GAA TFET in Figure 4c. With an increase in $V_{\mathrm{gs}}$ to 0.5 V, BTBT generation for silicon is near saturation and the around-source germanium is dominant resulting in the highest level of BTBT generation being 7.461e+29cm${}^{-3}$s${}^{-1}$ in Figure 4e. For the effectiveness of line tunnelling, a certain number of electrons are required (to form a virtual p-n junction) in the direction of the gate electric field. As shown in Figure 5a,c, a large volume of the channel region gets inverted, reducing the effective p-region at the virtual p-n junction at the gate interface. Therefore, line tunnelling occurs at the source, where the inversion region is not formed, as shown in Figure 4d,f [17]. Because the germanium-around-source structure changes the electric field, the around-source germanium region is also inverted in Figure 5b. This triggers line tunnelling in the silicon area at the boundary with the germanium layer and causes additional line BTBT tunnelling in the silicon area. Those phenomena of around-source germanium becoming dominant and the occurrence of line tunnelling give rise to enhanced tunnelling when BTBT generation for silicon reaches saturation, apart from suppressing the SS degradation behaviour and making $SS_{\mathrm{AVG}}$ of the GAS GAA TFET steadier compared with the Si GAA TFET and Ge-source GAA TFET. They also improve $I_{\mathrm{ON}}$ compared with the Si GAA TFET.

### 3.3. Optimised GAS GAA TFET

**Figure 6:**
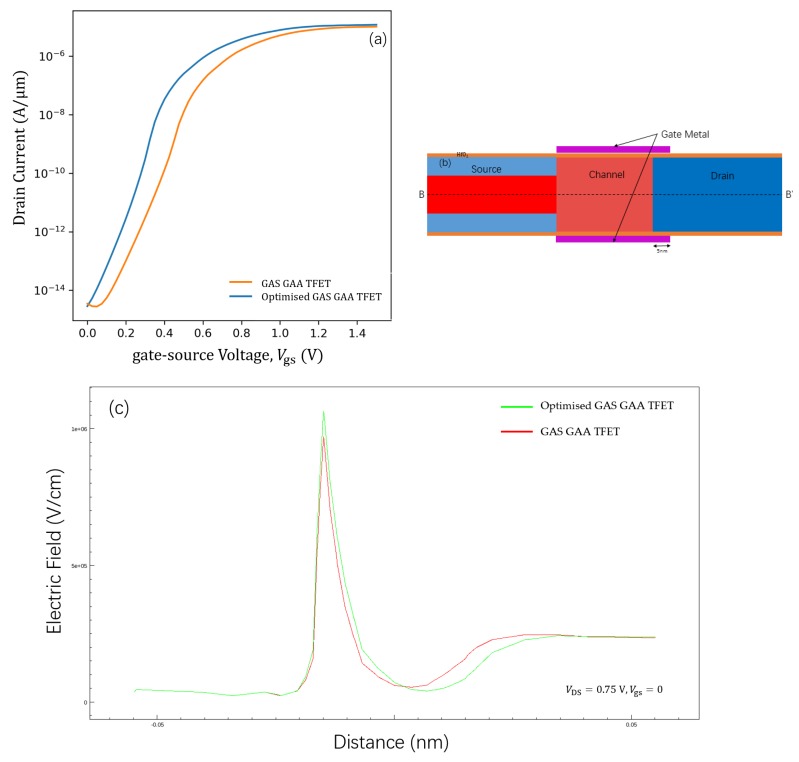
(**a**) $I_{D}-V_{\mathrm{gs}}$ transfer characteristics for the optimised and GAS GAA TFETs. (**b**) A cross-sectional view of the optimised GAS GAA TFET with a 5 nm drain overlapping; BB’ represents a cut line. (**c**) The electric field along the cut-line BB’ shown in (**b**).

With a decrease in the tunnelling length between the channel and the drain, the number of electrons that can tunnel from the valence band of the channel into the conduction band of the drain increases. This section discusses the use of work function [32] and drain overlapping engineering [33,34] for suppression of the ambipolar conduction effect on the GAS GAA TFET performance (Figure 2). Figure 6a shows the $I_{D}-V_{\mathrm{gs}}$ transfer characteristics of the GAS GAA TFET and optimised GAS GAA TFET. A schematic of the GAS GAA TFET with drain overlapping is shown in Figure 6b. Except for the gate material work function, which was tuned to 4.4 eV, and the 5 nm overlapping drain, there was no difference between the optimised GAS GAA TFET and the GAS GAA TFET at $T_{\mathrm{Ge}}$ = 6 nm. The optimised structure had a lower electric field at the channel and drain interface, as shown in Figure 6c. The lower electric field reduced the tunnelling probability at the channel and drain interface. The decrease in the tunnelling probability in turn reduced the ambipolar behaviour. In the transfer characteristics of the optimised GAS GAA TFET and GAS GAA TFET in Figure 6a, it is evident that the ambipolar behaviour of the former is alleviated. This results in the transfer characteristics of the optimised GAS GAA TFET being almost linear from $V_{\mathrm{gs}}$ = 0 V. The optimised GAS GAA TFET shows superior performance such as a steeper $SS_{\mathrm{AVG}}$ and a lower $I_{\mathrm{OFF}}$, apart from reduced ambipolar behaviour. A comparison of the optimised GAS GAA TFET with the GAS GAA TFET, Si GAA TFET, and Ge-source GAA TFET in terms of $SS_{\mathrm{MIN}}$, $SS_{\mathrm{AVG}}$, $I_{\mathrm{ON}}$, and $I_{\mathrm{OFF}}$ is presented in Table 2.

### 3.4. Process Flow

**Figure 7:**
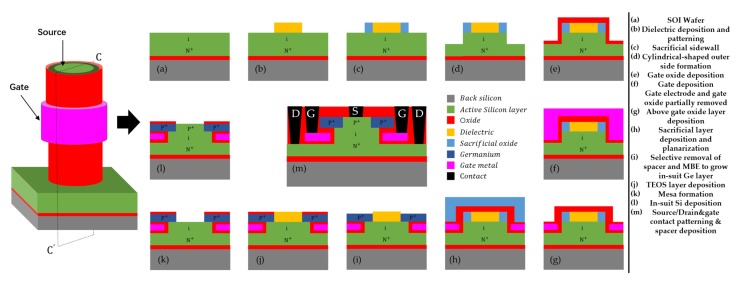
Fabrication process flow of a GAS GAA TFET along the cross section CC’.

Figure 7 summarizes the suggested fabrication processes for the GAS GAA TFET. The processes start with the formation of a cylindrical-shaped outer silicon layer via etching using electron beam lithography (EBL) followed by sacrificial sidewall deposition in Figure 7a–d [1]. The radius of dielectric, after deposition and patterning, determines the thickness of silicon and germaniums ($r=\frac{1}{2}T_{\mathrm{Si}}+T_{\mathrm{Ge}}$) in Figure 7b. Figure 7e shows the deposition of gate oxide. Figure 7f,g depict depositing a gate electrode on gate oxide layers and both gate electrode and gate oxide layer are partially removed, and then above gate oxide layer is deposited to form a spacer [35]. Afterwards, a sacrificial layer surrounding the above gate oxide layer is deposited followed by planarization in Figure 7h. Figure 7i illustrates the selective removal of the above gate oxide layer and the sacrificial layer followed by molecular beam epitaxy (MBE) to grow an in-situ boron-doped Ge layer as the around-source germanium [36]. Figure 7j depicts a TEOS layer deployed and planarized. Figure 7k shows all the layers exposing to the mesa [35]. Moreover, the in-suit boron-doped silicon is deposited as the germanium-around-source in Figure 7l. Finally, contacts and metal are formed for accessing the source, drain and gate in Figure 7m.

## 4. Conclusions

In this paper, we propose a novel GAS GAA TFET, with a steady and steeper $SS_{\mathrm{AVG}}$ and a higher $I_{\mathrm{ON}}$ than conventional TFETs such as the Si GAA TFET and Ge-source GAA TFET. The use of a germanium-around-source configuration and a combination of two materials with different bandgaps in the source suppressed the SS degradation, made $SS_{\mathrm{AVG}}$ steady and steeper compared with the Ge-source GAA TFET, and resulted in $I_{\mathrm{ON}}$ being higher than that of the Si GAA TFET. The effect of an increase in the thickness of the germanium layer on $I_{\mathrm{ON}}$ was investigated. Furthermore, the ambipolar behaviour of the GAS GAA TFET could be alleviated through work function and drain overlapping engineering. The optimised GAS GAA TFET showed a steady and steep $SS_{\mathrm{AVG}}$ of 57.29 mV/decade, a significantly high $I_{\mathrm{ON}}$ of 11.9 μA and a low $I_{\mathrm{OFF}}$ of $2.85\times 10^{-9}$μA, and absence of ambipolar behaviour. These features indicate the high potential of the device for use in low-power applications.

## Figures and Tables

**Table 1:** Parameters used for the Synopsys Sentaurus technology computer-aided design (TCAD) simulation.

Parameters	Definations	Value
*r*	Device radius	12 nm
$L_{\mathrm{SD}}$	Lateral length of source and drain	40 nm
$L_{C}$	Lateral length of channel	30 nm
$T_{\mathrm{ox}}$	Gate oxide thickness	2 nm
$T_{\mathrm{Ge}}$	Thickness of around-source germanium	Variable
$T_{\mathrm{Si}}$	Thickness of silicon surrounded by germanium	Variable
$N_{S}$	P-type source doping concentration	$5\times 10^{19}\;$cm${}^{-3}$
$N_{C}$	P-type channel doping concentration	$1\times 10^{15}\;$cm${}^{-3}$
$N_{\mathrm{SGe}}$	P-type around-source germanium doping concentration	$5\times 10^{19}\;$cm${}^{-3}$
$N_{D}$	N-type drain doping concentration	$1\times 10^{17}\;$cm${}^{-3}$

**Table 2:** A comparison of optimised GAS GAA TFET with GAS GAA TFET, Si GAA TFET, and Ge-source GAA TFET.

	Si GAA TFET	Ge-Source GAA TFET	GAS GAA TFET	Optimised GAS GAA TFET
$SS_{\mathrm{MIN}}$ (mV/dec.)	26.835	58.645	45.720	39.501
$SS_{\mathrm{AVG}}$ (mV/dec.)	68.71	83.71	65	57.29
$I_{\mathrm{ON}}$ (μA/um)	$9.38\times 10^{-4}$	11.7	10.2	11.9
$I_{\mathrm{OFF}}$ (μA/um)	$5.05\times 10^{-13}$	$1.722\times 10^{-8}$	$3.49\times 10^{-9}$	$2.85\times 10^{-9}$

## Abbreviations

The following abbreviations are used in this manuscript besides parameters in Table 1:

BTBT	band-to-band tunnelling
$T_{\mathrm{WKB}}$	band-to-band tunnelling probability
$E_{g}$	the bandgap of main semiconductor material in device
GAS GAA TFET	germanium-around-source gate-all-around tunnel field-effect transistor
Si GAA TFET	silicon gate-all-around tunnel field-effect transistor
Ge-source GAA TFET	germanium-based-source gate-all-around tunnel field-effect transistor
SS	subthreshold swing
$SS_{\mathrm{MIN}}$	minimum point subthreshold swing
$SS_{\mathrm{AVG}}$	average subthreshold swing
$I_{\mathrm{ON}}$	ON-state current
$I_{\mathrm{OFF}}$	OFF-state current
$I_{D}$	drain current
$I_{T}$	current at $V_{T}$
$V_{\mathrm{ON}}$	the voltage where OFF-state current increased by a factor of $10^{7}$ times
$V_{\mathrm{ONSET}}$	the voltage after which the drain current increases exponentially with the gate voltage
$V_{\mathrm{DS}}$	drain-source voltage
$V_{\mathrm{gs}}$	gate-source voltage
$V_{T}$	threshold voltage
EBL	electron beam lithography
MBE	molecular beam epitaxy
TEOS	Tetraethylortho Silicate
